# Medicine shortages and glaucoma care: a time-series analysis indicating population-level consequences of a national timolol eye drop shortage

**DOI:** 10.1007/s11096-026-02209-w

**Published:** 2026-08-08

**Authors:** Erin Fawcett, Lisa Kalisch Ellett, Simi Sarin, Cassandra Haines, Helen Stone, Jack Janetzki

**Affiliations:** 1 Adelaide University, Adelaide, South Australia Australia; 2 Flinders University, Bedford Park, GPO Box 2100, Adelaide, South Australia Australia; 3 Pharmaceutical Society of Australia, Suite 7/102 Greenhill Rd, Unley, South Australia Australia

**Keywords:** Drug substitution, Glaucoma, Optometry, Pharmacy, Pharmacy research, Policy, Timolol

## Abstract

**Introduction:**

Medicine shortages can disrupt glaucoma management and necessitate therapeutic substitution. Understanding how shortages affect access to first-line treatments for glaucoma is important for optometrists when monitoring intraocular pressure control, and pharmacists when counselling patients and coordinating care during periods of limited medicine availability. Timolol, a non-selective beta-blocker, is a first-line treatment for glaucoma and ocular hypertension and has experienced international shortages in recent years. Between August 2024 and May 2025, Australia experienced a nationwide shortage of timolol-containing eye drops, raising concerns regarding continuity of care, access to treatment, and therapeutic substitution.

**Aim:**

To descriptively examine the impact of the 2024–2025 timolol eye drop shortage on dispensing patterns of antiglaucoma medicines in Australia and assess the contribution of imported products in mitigating the shortage.

**Method:**

A retrospective drug utilisation study was conducted using publicly available aggregate Pharmaceutical Benefits Scheme (PBS) and Repatriation PBS dispensing data from January 2020 to August 2025. Monthly dispensings of antiglaucoma preparations (ATC S01E) were standardised per 100,000 population and analysed using descriptive time-series methods. Dispensing trends were compared before, during, and after the shortage, with stratification by timolol formulation, therapeutic class, and product type (locally supplied versus overseas imports).

**Results:**

The late 2024 and early 2025 timolol shortage was associated with a sustained decline in dispensing of single-ingredient extended-release timolol, decreasing from approximately 40 dispensings per 100,000 population per month before the shortage to 1.07 by March 2025. Dispensing of standard-release timolol increased temporarily in October 2024 before declining in a similar pattern. Overseas imports accounted for 24.3% of all timolol dispensings during the shortage period and peaked at 87% of timolol dispensings in March 2025. Despite regulatory approval of imported products, overall timolol utilisation remained below pre-shortage levels. Alternative therapeutic classes, including prostaglandin analogues and carbonic anhydrase inhibitors, showed modest increases in dispensing, suggesting potentially limited therapeutic substitution.

**Conclusion:**

The timolol shortage disrupted access to a cornerstone first-line treatment for glaucoma. Regulatory intervention through the approval of imported products partially mitigated the shortage but did not restore utilisation to pre-shortage levels in Australia. Future shortage management strategies should prioritise rapid policy activation and closer alignment of imported products with formulations routinely available in Australia.

**Supplementary Information:**

The online version contains supplementary material available at https://doi.org/10.1007/s11096-026-02209-w.

## Impact Statements

- This study provides evidence on how a timolol shortage affected access to a first-line glaucoma therapy in Australia and the role of regulatory interventions in maintaining medicine availability.
- The findings highlight the importance of pharmacists and optometrists recognising medicine supply disruptions early, supporting timely therapeutic substitution, and coordinating care to minimise interruptions to glaucoma management.
- The limited recovery of timolol utilisation despite imported product availability demonstrates the need for proactive shortage planning, improved alignment of alternative products with routine clinical use, and rapid implementation of mitigation strategies.

## Introduction

Timolol, a non-selective beta-blocker, is widely prescribed as monotherapy or combination therapy for reducing elevated intraocular pressure (IOP) in glaucoma and ocular hypertension [1–3]. In advanced glaucoma, even brief interruptions to therapy may accelerate irreversible vision loss, highlighting the importance of appropriate management during medicine shortages [4]. Medicine shortages can affect continuity of care, leading to treatment interruption, therapeutic substitution, and potential adverse clinical outcomes [5].

Internationally, ophthalmic medicine shortages have been associated with treatment changes, increased healthcare burden and disrupted continuity of care [6–9]. However, evidence regarding their population-level impact and the effectiveness of mitigation strategies remains limited.

Between August 2024 and May 2025, Australia experienced a nationwide shortage of timolol-containing eye drops, both in single-ingredient and some combination formulations [10]. Shortages of timolol products were officially recognised by the Therapeutic Goods Administration (TGA), the national regulator of medicines in Australia, on November 29, 2024 for the standard, single ingredient formulation, and on August 12, 2024 for the single ingredient extended-release formulation [10]. The TGA communicated shortage information, including expected duration, contributing factors, and management recommendations, through public shortage notifications and the Medicine Shortage Reports Database [10].

Normally, only medicines registered on the Australian Register of Therapeutic Goods (ARTG) [11] can be routinely supplied in Australia, with unregistered overseas products requiring regulatory pathways such as the Special Access Scheme [12] or Section 19A (S19A) approval. During the timolol shortage, from November 1, 2024, to June 30, 2025, three overseas products were imported under S19A of the Therapeutic Goods Administration Act 1989 [13]. S19A permits the temporary importation of overseas-approved medicines for urgent supply during shortages to ensure continuity of treatment [14]. Three S19A timolol products (two extended-release and one standard-release formulation) were made available during the shortage [13].

At approximately the same time as the timolol shortages, Australian legislation for 60-day dispensing of medicines was introduced. This enabled prescribers to issue prescriptions under Australia’s Pharmaceutical Benefits Scheme, the nationally subsidised medicine formulary, for 60-day supplies rather than the existing 30-day supply for certain medications [15]. Ophthalmological preparations were introduced in Stage 3 on 1 September 2024, occurring during the extended-release timolol shortage and shortly before the standard-release shortage. This temporal overlap raised concerns that increased quantities supplied per prescription may influence medicine availability during periods of constrained supply.

### Aim

To descriptively examine the impact of the 2024–2025 timolol eye drop shortage on dispensing patterns of antiglaucoma medicines in Australia and assess the contribution of imported products in mitigating the shortage.

## Method

A retrospective study utilising descriptive time series analysis was conducted using Pharmaceutical Benefits Scheme (PBS) and Repatriation Pharmaceutical Benefits Scheme (RPBS) Section 85 and Section 100 Date of Supply Data [16]. The PBS is Australia’s national medicines formulary and the RPBS is the national formulary for veterans, war widows or widowers and their dependents based on several eligibility criteria [17]. This dataset contains population-level dispensings of most medicines subsidised by the Australian Government. Monthly dispensings of medicines are aggregated and reported by item code (the pharmacy reimbursement code). This dataset was linked to the PBS item drug map (October 2025 version) to identify specific medicines, strengths and formulations, and S19A products based on medicine names of each product [16]. S19A products available in Australia but not PBS-listed are not captured in this dataset. The study period was defined as January 2020 to August 2025 providing a long baseline of pre-shortage dispensings and adequate follow-up after the 2024 shortage.

Shortage data for ophthalmic products were obtained from the Active and Archived Medication Shortages lists from the Therapeutic Goods Administration website [10].

### Medicines of interest

Medicines classified under the Anatomical Therapeutic Classification (ATC) code “S01E Antiglaucoma preparations and miotics” were included in the primary analysis where PBS-listed during the study period (Appendix 1).For single-ingredient timolol analyses, dispensings of PBS-listed items classified as “S01ED01” were investigated. To assess changes in ophthalmic beta-blocker use during timolol shortages, dispensing patterns of “S01ED—beta blocking agents” and timolol-containing “S01EA, S01EC, S01EE” combination products were examined (Appendix 1).

### Analytic approach

All dispensings of medicines beginning with an Anatomical Therapeutic Classification code of “S01E” were extracted from the dataset by the month and year in which they occurred. The dispensings per month were then plotted as time series for timolol single-ingredient preparations, beta-blockers and all “S01E” medicines. National quarterly Estimated Residential Population estimates from the Australian Bureau of Statistics were used to calculate the number of dispensings per 100,000 population per month allowing for visualisation of dispensing trends over time at a population-level [18]. Over the study period, the mean number of dispensings each month, and the mean number of dispensings per 100,000 population each month over the study period was calculated. Mean dispensings before, during and after shortages were compared for ophthalmic preparations to describe population-level changes that occurred including population-level switching to alternative ophthalmic preparations. For timolol single-ingredient preparations, there are two products available on the market in Australia: standard-release (conventional) and extended-release (gel-forming). The standard-release drops are given once or twice a day whereas the extended-release drops are only administered once a day. For this reason, it is hypothesised that people who are using standard-release timolol may be able to switch to extended-release drops during a period of shortage and still be provided with the same duration of therapy.

The rate of dispensing for antiglaucoma and miotic ophthalmic preparations was calculated by using the slope function in Excel measuring the slope of best-fit line between the number of dispensings over the study period given the number of months between January 2020 and August 2025. The analysis was repeated at the therapeutic class-level analysis too. The proportion of single ingredient timolol dispensings that were dispensed as S19A products was also quantified between start of their dispensing in August 2024 to August 2025.

Dispensing of prescriptions with 60-day PBS item codes was accounted for by carrying over the dispensings from the first month into the second assuming that people would then have sufficient supply for two months of therapy. For example, a single 30-day prescription dispensing of timolol-containing eye drops would be counted in the first month but not the second, whereas a 60-day prescription dispensing of timolol-containing eye drops would be counted in both the first and second month. As such, the total dispensings in a month would include some 30-day and some 60-day dispensings of prescriptions. A simplified example is provided in Table 1.

**Table 1:** Example of how 30-day PBS items and 60-day PBS item dispensings are included in a time series analysis

	30-day timolol prescription	60-day timolol prescription (single prescription with first dispensing in July)	Total timolol prescription dispensings per month
July 2024	1	1	2
August 2025	0	1	1

PBS data do not contain person-level treatment duration or adherence information and therefore dispensing events were used as a proxy for medicine utilisation. Time series graphs were annotated with data from the Therapeutic Goods Administration medicine shortages database to assist in visualisation of when shortages occurred. In doing so the impact of the 2024 timolol shortage on dispensing volumes of timolol and other IOP-lowering preparations was able to be quantified. Trends in the use of alternative intraocular pressure-lowering medicines were constructed for the same period to allow observation of any compensatory changes in use associated with the timolol shortage and other potential substitution behaviours at a population level. Shortages in the use of alternative IOP-lowering medicines were also identified from the Therapeutic Goods Administration medicine shortages database.

### Ethics approval

The project uses publicly available, de-identified Pharmaceutical Benefits Scheme aggregate data. As no individual-level data are used, ethics approval was not required.

## Results

**Fig. 1:**
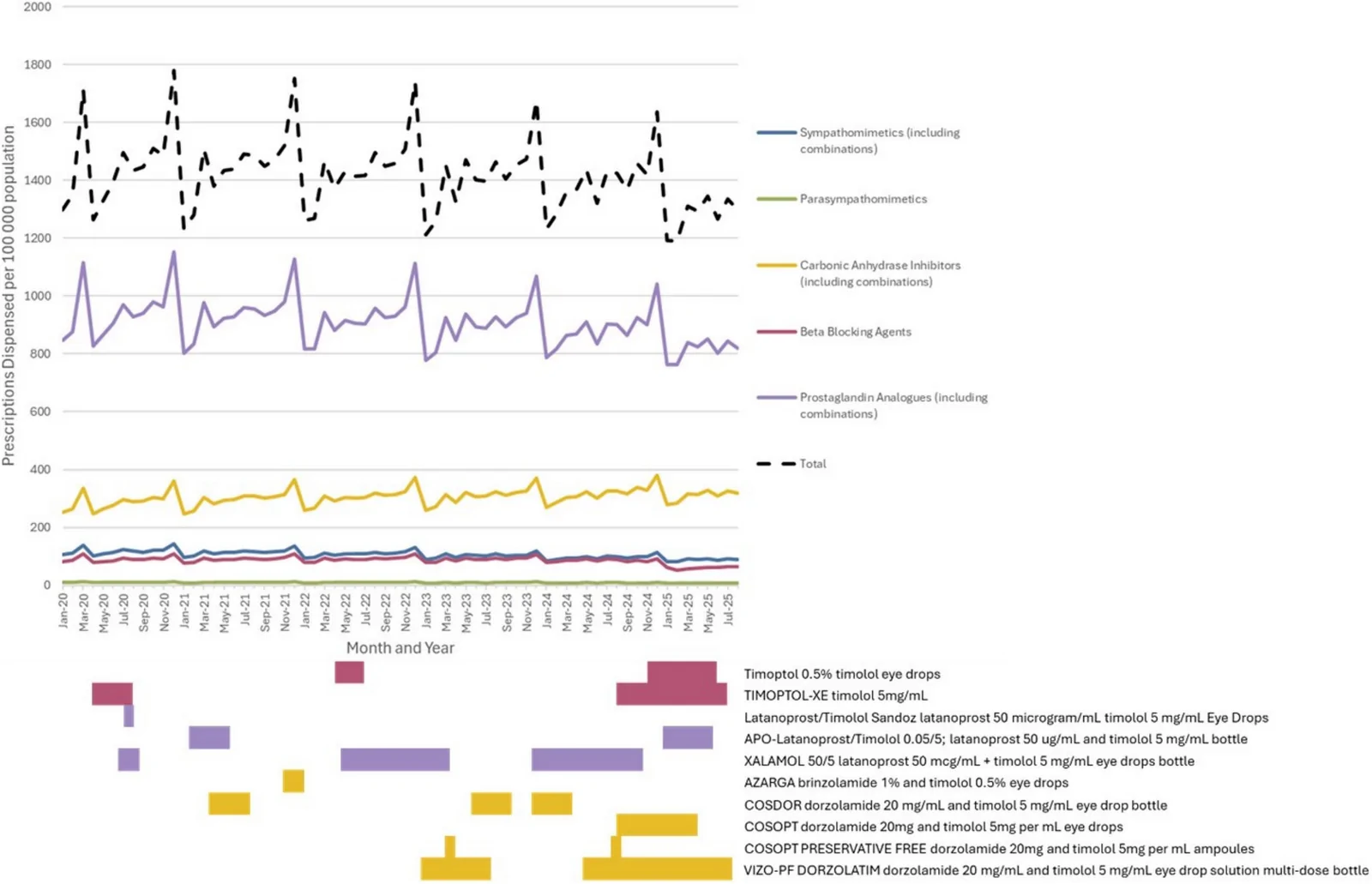
Antiglaucoma preparations and miotics prescription dispensed per 100,000 population per month between January 2020 and August 2025 by fourth level Anatomical Therapeutic Classification code. Coloured blocks represent shortages of corresponding timolol-containing products

Between January 2020 and August 2025 there was a mean number of 1414 antiglaucoma preparations and miotics dispensed/100,000 population/month (Fig. 1). The main class of ophthalmic preparations dispensed during the study period was prostaglandin analogues (including combination products), with a mean number of 907 dispensings/100,000 population/month. This was followed by carbonic anhydrase inhibitors (including combinations), with a mean of 305 dispensings/100,000 population/month over the study period. For sympathomimetic ophthalmic preparations, the mean number of dispensings/100,000 population/month was 106, whereas for beta-blocker agents it was 87. Parasympathomimetic products were the least frequently dispensed antiglaucoma preparations, with a mean of 10 dispensings/100,000 population/month during the study period.

The rate of dispensing for antiglaucoma medicines changed over the study period. For overall antiglaucoma and miotic ophthalmic preparations, the dispensing rate decreased by 1.59 dispensings/100,000 population/month. At the class level, the rate of dispensing of prostaglandin analogues decreased by 1.42 dispensings per 100,000 population/month, sympathomimetics decreased by 0.44 dispensings/100,000 population/month, beta-blocking agents decreased by 0.28 dispensings/100,000 population/month and parasympathomimetics decreased by 0.04 dispensings/100,000 population/month. The rate of dispensing for carbonic anhydrase inhibitors (including combinations), however, increased by 0.59 dispensings/100,000 population/month.

During the study period, there were also intermittent shortages of several ocular medicines. In late 2024, however, multiple ocular medicines experienced shortages, including timolol, latanoprost/timolol and dorzolamide. Appendix 2 includes a full list of these shortages from the TGA medicines shortage database archive report for the full study period.

**Fig. 2:**
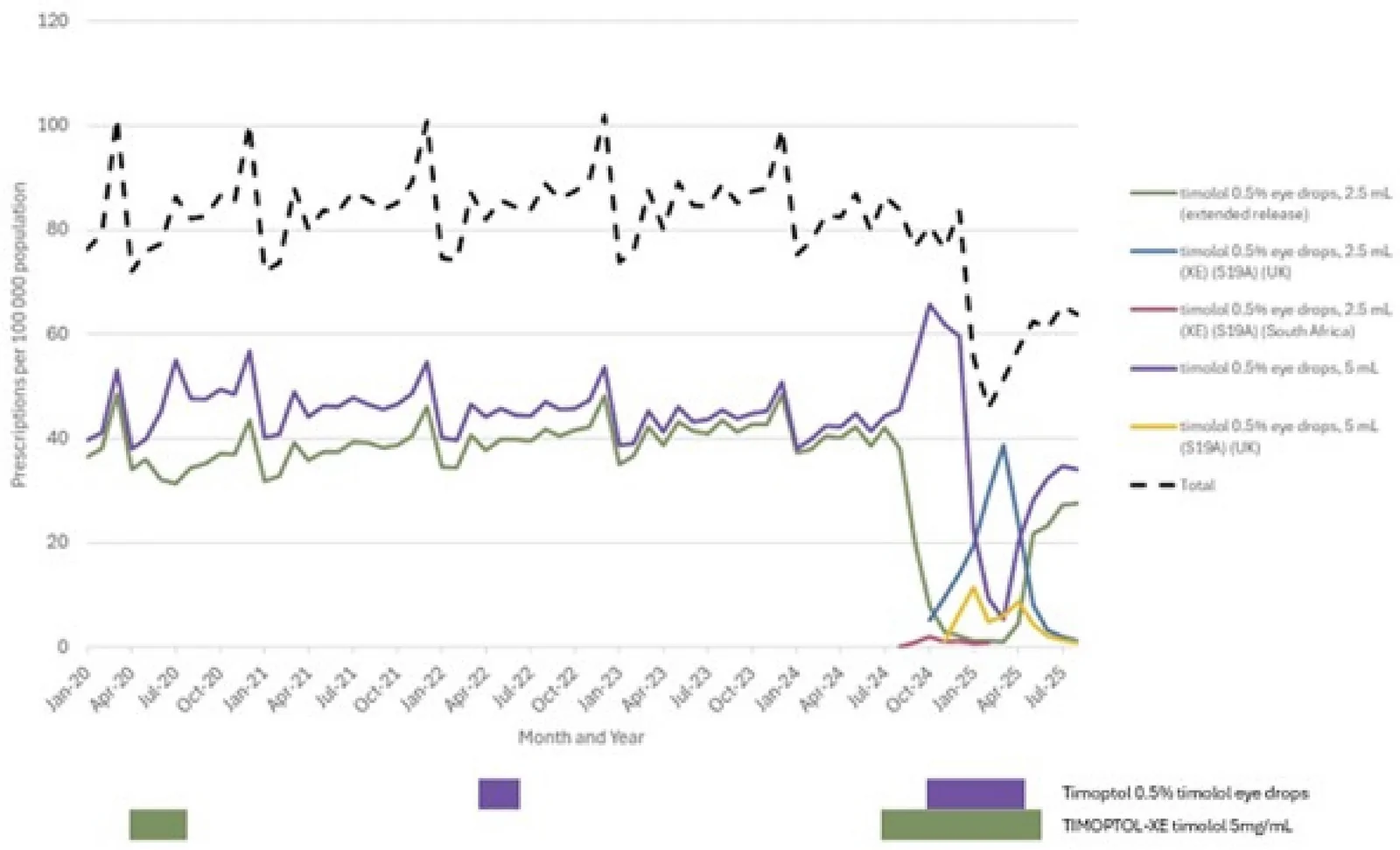
Single ingredient timolol prescriptions dispensed per month per 100,000 population by item and formulation. Coloured blocks represent shortages of corresponding timolol-containing products

Overall, single ingredient timolol (Fig. 2) had a mean of 81 dispensings/100,000 population/month throughout the study period. For standard-release 0.5% timolol, the mean number of dispensings was 44/100,000 population/month and extended-release 0.5% timolol eye drops had a mean number of 34 dispensings/100,000 population/month across the study period. Before the single-ingredient timolol shortages in mid-2024, timolol 0.5% eye drops, 5 mL, were the most frequently dispensed formulation, followed by timolol 0.5% eye drops, 2.5 mL (extended-release). As the timolol 0.5% eye drops, 2.5 mL (extended-release) shortage occurred (August 2024 to June 2025), prescriptions/100,000 population/month fell first for timolol 0.5% eye drops, 2.5 mL (extended-release), from approximately 40 dispensings/100,000 population/month in July 2024 to a low of 1.07 by March 2025. In the early months of the same shortage, there was a subsequent rise in prescriptions dispensed/100,000 population/month for timolol 0.5% standard release eye drops 5 mL, peaking at 65.69 dispensings/100,000 population/month in October 2024 before also experiencing a shortage (November 2024 to May 2025) and falling to a low of 5.42 dispensings/100,000 population/month in March 2025.

During the mid-2024 timolol shortages, three S19A timolol products were made available timolol 0.5% 2.5 mL extended-release (Timoptol-LA) from the United Kingdom; timolol 0.5% 2.5 mL extended-release (Timoptol XE) from South Africa; and timolol 0.5% 5 mL standard-release (Timoptol) from the United Kingdom. During the late-2024 timolol shortage, non-extended-release S19A timolol drops were available immediately; however, the extended-release formulation was only first dispensed in October 2024. The greatest utilisation of an S19A product during the study period was observed for the timolol 0.5% 2.5 mL extended release (UK) product, with a mean number of dispensings/100,000 population/month of 14.09 during its period of availability. In contrast, for timolol 0.5% 5 mL standard-release eye drops (UK) and timolol 0.5% eye drops, 2.5 mL extended-release (South Africa), the mean number of dispensings/100,000 population/month was 4.76 and 1.00, respectively, during their availability. By the end of the study period, despite resolution of the timolol shortages, overall dispensings of single ingredient timolol had not returned to pre-shortage levels.

**Fig. 3:**
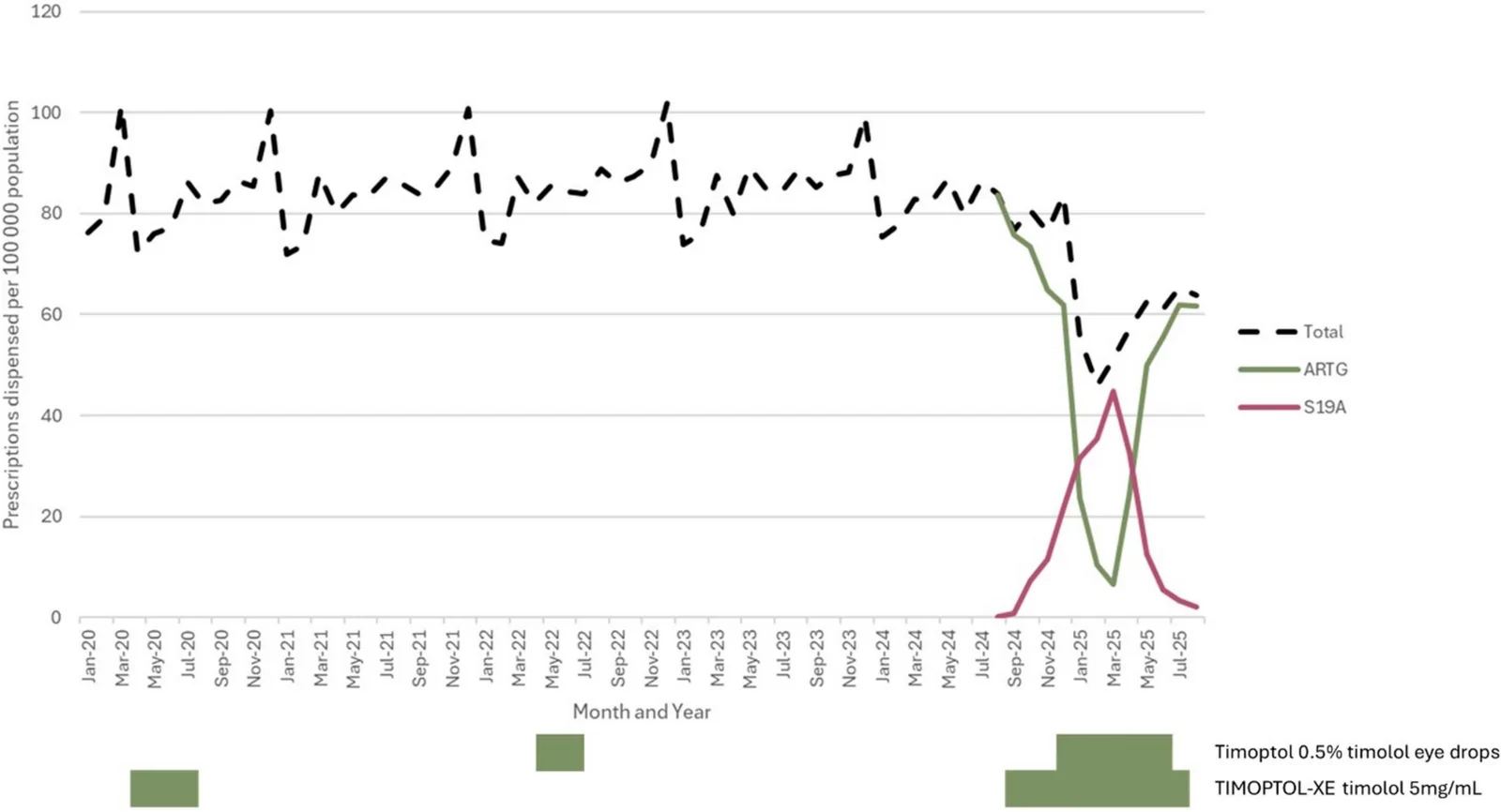
Single ingredient timolol prescriptions dispensed per month per 100,000 population by Australian Register of Therapeutic Goods or S19A Pharmaceutical Benefits Scheme-listed listing. Coloured blocks represent shortages of corresponding timolol-containing products

Figure 3 shows the overall dispensing of single ingredient timolol prescriptions per 100,000 population per month across the study period by Australian Register of Therapeutic Goods or S19A listing. S19A products accounted for 24% of all single-ingredient timolol dispensings between August 2024 and August 2025, exceeding 50% of dispensings between February and April 2025 and peaking at 87% in March 2025 (Fig. 3).

**Fig. 4:**
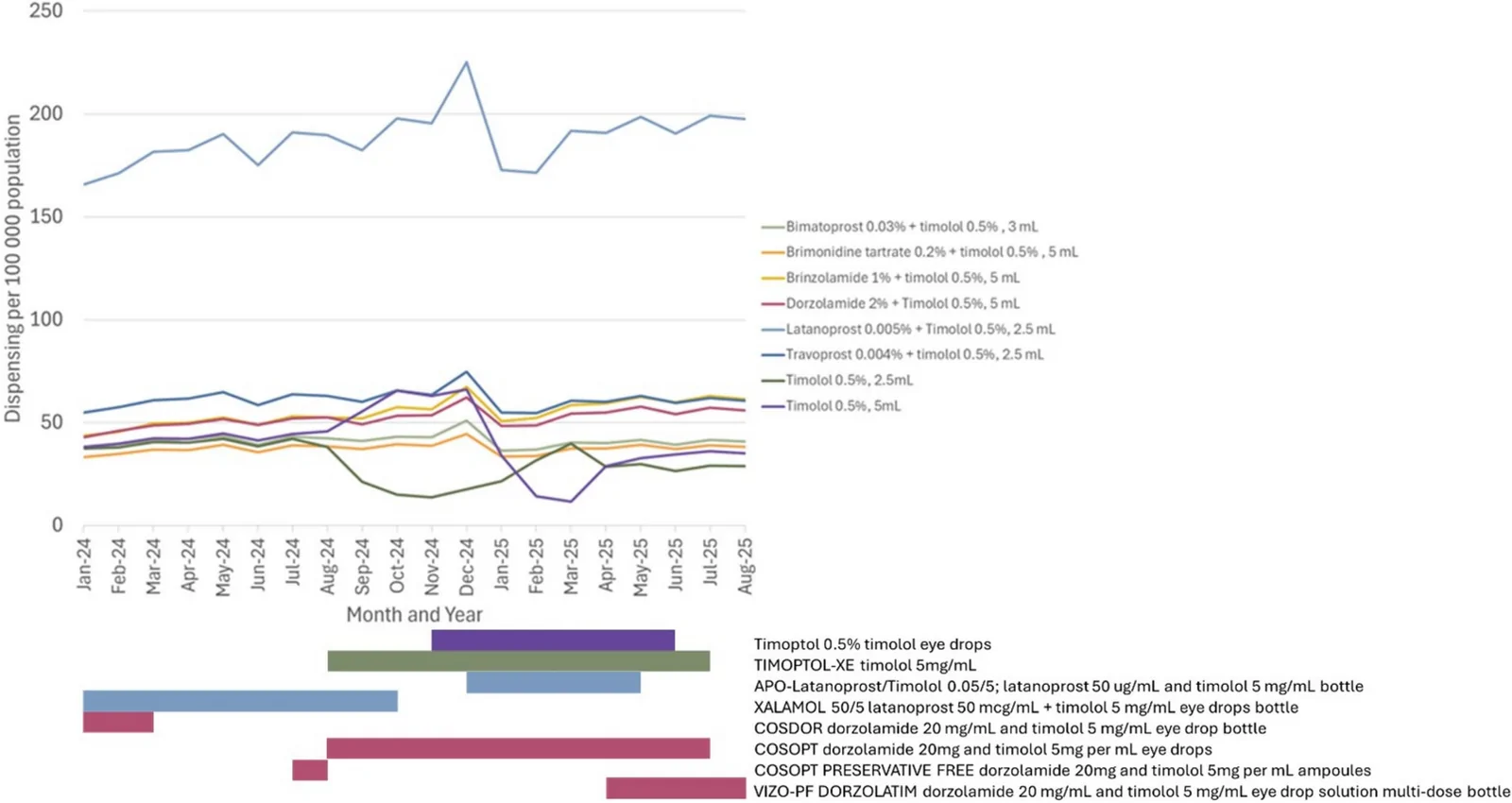
All timolol containing eye drops dispensed per 100,000 population per month by product. Coloured blocks represent shortages of corresponding timolol-containing products

When observing dispensings of both single-ingredient and multiple-ingredient preparations that contain timolol, there were mainly shifts observed in the dispensing patterns of single-ingredient timolol products (Fig. 4 and as above in Fig. 2), but no great change was observed for multiple-ingredient preparations (Fig. 4).

**Fig. 5:**
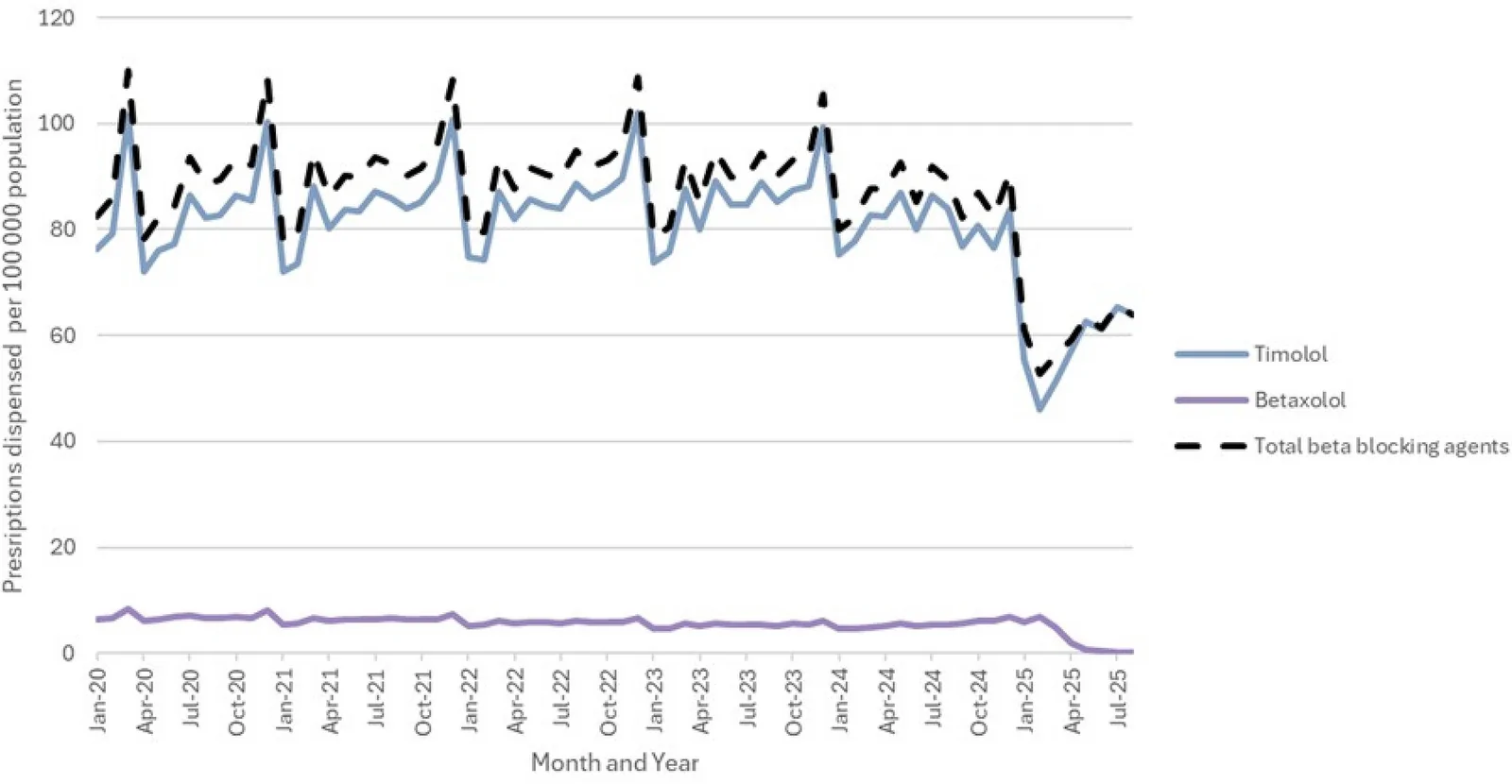
Beta-blocking agents: single-ingredient timolol and single-ingredient betaxolol product dispensings per 100,000 population

Within class comparison of monthly dispensings of ocular beta-blockers per 100,000 population/month (Fig. 5) also demonstrated no great population-level change from dispensing of timolol-containing products to betaxalol. Betaxolol was deleted from the Pharmaceutical Benefits Scheme and Australian market in May 2025.

## Discussion

Pharmacists routinely manage medicine shortages in practice, however, there is little population-level evidence quantifying how shortages affect utilisation of ophthalmic medicines or whether regulatory mitigation strategies maintain access.

This study demonstrates that the 2024–2025 timolol shortage in Australia resulted in a substantial decline in single-ingredient timolol dispensings, only partially mitigated by S19A PBS-listed imports and substitution with available alternatives. Although S19A PBS-listed imports accounted for more than half of timolol dispensings during the shortage, overall utilisation of timolol-containing products remained below pre-shortage levels by end of the study.

Mirroring standard practice, during the timolol shortage pharmacists were unable to independently substitute timolol-containing products unless it was from an ARTG-registered PBS product to an equivalent S19A PBS-listed product. Any other substitution, for example, strength, formulation or providing the individual products of a combination product separately required consultation with the prescriber. Advice from national and state-level medicine regulators and professional peak bodies was variable and predominantly limited to logistical information such as duration of supply and brief details on reason for the shortage [10, 19].

Dispensing trends indicated a sequential disruption, with extended-release formulations affected first, followed by standard-release products. Temporary increases in standard-release 5 mL dispensings suggest initial switching to available alternatives; however, this strategy likely became unsustainable once supply constraints extended across formulations. S19A PBS-listed products, particularly extended-release formulations, supported continuity of therapy and became the predominant products during the peak shortage period. Nevertheless, imported products represented only a proportion of expected utilisation, suggesting that S19A supply alone was insufficient to fully restore access. Contributing factors may have included limited formulation availability, constrained import volumes, and uncertainty regarding alternative products.

### Role and limitations of S19A

The effectiveness of S19A PBS-listed medicines depends on timeliness of implementation and alignment with clinical practice [20]. In this shortage, extended-release S19A products became available within one month of shortage recognition, demonstrating a rapid regulatory response. However, equivalent products were not available for all formulations, and imported medicines did not always align with routinely prescribed Australian products, potentially limiting prescriber confidence and patient acceptance.

Despite S19A availability, timolol utilisation remained below baseline levels. While dispensing data cannot determine causality, incomplete formulation equivalence and delays in pharmacy procurement may have contributed. Unlike routinely stocked medicines, S19A products are generally sourced after prescription receipt, introducing additional delays that may discourage treatment continuation. Therefore, regulatory approval alone may not ensure timely patient access.

### Substitution patterns

Timolol-containing combination products demonstrated greater resilience than single-ingredient products, with relatively minor dispensing changes during the shortage. Limited changes in prostaglandin analogue and carbonic anhydrase inhibitor dispensing suggest that broad therapeutic substitution was uncommon. This is clinically important because alternative glaucoma therapies have distinct safety considerations. Topical beta-blockers and carbonic anhydrase inhibitors may undergo systemic absorption, increasing risks of adverse effects in patients with respiratory, cardiovascular, or renal comorbidities [21, 22]. Although prostaglandin analogues are first-line therapies with generally favourable safety profiles, they may cause irreversible hyperpigmentation, hyperaemia, and eyelash growth [23]. Given that glaucoma predominantly affects older adults with substantial comorbidity burden, therapeutic substitution requires careful consideration. Patients receiving specific timolol regimens may therefore experience challenges maintaining continuity of therapy during shortages.

Seasonal dispensing patterns and PBS safety net effects contributed to temporary increases in utilisation but did not represent sustained recovery. The increase observed in December 2024 likely reflected patients reaching annual PBS safety net thresholds before reset on 1 January [24]. Limited use of 60-day dispensing for single-ingredient timolol reduced the likelihood that extended supply intervals influenced observed trends.

International evidence highlights the broader vulnerability of ophthalmic medicines to shortages. A study of a 2018 dorzolamide/timolol shortage in the United States reported that clinically appropriate substitution maintained intraocular pressure control but increased administrative burden [8]. Similarly, recurrent ophthalmic shortages in the United States have disrupted clinical workflows and increased costs for patients [25]. A recent analysis identified 379 ophthalmic medicine shortages between 2001 and 2024, with shortages frequently prolonged and associated with manufacturing and supply–demand issues [7]. These findings align with the current study, demonstrating that mitigation strategies can provide partial rather than complete resilience [25].

### Strengths and limitations

A major strength of this study is the use of national PBS aggregate data over five years, enabling assessment of baseline utilisation, shortage disruption, and mitigation strategies. Classification by Anatomical Therapeutic Classification code and differentiation between ARTG-registered and S19A PBS-listed products enabled interpretation of potential substitution behaviours.

Private (non-PBS) supply of medicine was not captured, potentially underestimating timolol utilisation, particularly before S19A PBS-listed product availability. Shortage timing was inferred from public reports and dispensing changes, which may not precisely reflect local product availability. Seasonal PBS safety net effects may also have influenced apparent recovery in utilisation towards end of the year [4, 24].

Although interrupted time series analysis could provide statistical estimates of changes associated with the shortage, this was not undertaken because aggregate dispensing data for ophthalmic preparations are difficult to interpret in the context of variable treatment duration and formulation longevity. Descriptive time-series analysis was therefore considered the most appropriate approach for characterising national dispensing patterns during the shortage.

Dispensing data cannot fully capture clinical consequences. Differences in formulation characteristics may affect tolerability and adherence; for example, gel-forming timolol formulations reduce dosing frequency but may cause transient visual disturbance [26]. Substitution may also introduce class-specific adverse effects, including prostaglandin analogue-associated periorbitopathy [27].

Additionally, changes between unfixed and fixed combination therapies may influence intraocular pressure control, as unfixed regimens may provide superior diurnal control in some patients [28].

Importantly, continued medicine supply does not necessarily indicate stable disease control, as glaucoma progression requires structural and functional monitoring, including optical coherence tomography and visual field assessment [29].

### Policy and practice implications

These findings highlight the importance of proactive shortage management. Clinicians should anticipate formulation-specific vulnerabilities, establish switching plans, and collaborate with pharmacists and regulators to minimise disruption. Pharmacists have an important role in facilitating continuity of care by communicating S19A product availability and supporting patient counselling.

For policymakers, the timolol shortage demonstrates that regulatory responses require timely activation, alignment with clinical practice, and sufficient supply capacity. Future strategies should prioritise rapid S19A implementation, availability of clinically equivalent formulations, diversified supply pathways, and mechanisms such as Serious Scarcity Substitution Instruments to enable timely pharmacist-led substitution where appropriate [12]. Clear guidance regarding dose equivalence and communication strategies may improve uptake of alternative products.

### Future research

Future research should link dispensing data with clinical outcomes and explore patient, pharmacist and prescriber experiences to better understand responses to medicine shortages.

## Conclusion

The 2024–2025 timolol shortage disrupted access to a cornerstone therapy for glaucoma and ocular hypertension. While S19A timolol eye drop imports mitigated the impact, they did not fully restore overall timolol use to pre-shortage utilisation levels. Strengthening early S19A Pharmaceutical Benefit Scheme-listing approval, expanding product coverage, and improving operational guidance are critical to safeguarding continuity of care during future medicine shortages.

## Supplementary Information

Below is the link to the electronic supplementary material.

Supplementary file1 (DOCX 23 KB)

## Data Availability

Data to conduct this study were obtained from https://www.pbs.gov.au/info/statistics/dos-and-dop/dos-and-dop.
