# Supplementary material for: Medicine shortages and glaucoma care: a time-series analysis indicating population-level consequences of a national timolol eye drop shortage
Source: Int J Clin Pharm. 2026 Aug 8;48(6):2312–21. doi: 10.1007/s11096-026-02209-w (PMC13731515; doi:10.1007/s11096-026-02209-w)
Supplement: Supplementary file 1 [file 11096_2026_2209_MOESM1_ESM.docx]

**Title:**

Medicine shortages and glaucoma care: a time-series analysis indicating population-level consequences of a national timolol eye drop shortage

**Running title:**

Timolol Shortage and Glaucoma Care

**Authors:**

Erin Fawcett^a^, Lisa Kalisch Ellett^a^, Simi Sarin^b^, Cassandra Haines^b^, Helen Stone^c^, Jack Janetzki^a^

**Affiliations:**

^a^ Adelaide University, Adelaide, South Australia, Australia

^b^ Flinders University, Bedford Park, GPO Box 2100, Adelaide, South Australia, Australia

^c^ Pharmaceutical Society of Australia, Suite 7/102 Greenhill Rd, Unley, South Australia, Australia

**Contact author:**

Jack Janetzki; [Jack.Janetzki@adelaide.edu.au](mailto:Jack.Janetzki@adelaide.edu.au)

**Corresponding author name and email address**

Jack Janetzki Jack.Janetzki@adelaide.edu.au

**ORCiDs:**

Erin Fawcett: https://orcid.org/0009-0009-7100-4416

Lisa Kalisch Ellett: https://orcid.org/0000-0001-5063-6128

Simi Sarin: https://orcid.org/0009-0008-4350-423X

Cassandra Haines: https://orcid.org/0009-0009-4863-5228

Helen Stone: https://orcid.org/0009-0006-4915-0423

Jack L Janetzki: https://orcid.org/0000-0002-4171-3504

**Appendices:**

Appendix 1: Table of all medicines included in the analysis by Anatomical Therapeutic Classification Code, medicine name, form, strength and Pharmaceutical Benefits Scheme item code

S01EC01 ACETAZOLAMIDE Tablet 250 mg 01004W

S01ED01 TIMOLOL Eye drops 5 mg (as maleate) per mL, 5 mL 01279H

S01ED01 TIMOLOL Eye drops (gellan gum solution) 5 mg (as maleate) per mL, 2.5 mL 01926J

S01EB01 PILOCARPINE Eye drops containing pilocarpine hydrochloride 10 mg per mL, 15 mL 02595N

S01EB01 PILOCARPINE Eye drops containing pilocarpine hydrochloride 20 mg per mL, 15 mL 02596P

S01EB01 PILOCARPINE Eye drops containing pilocarpine hydrochloride 40 mg per mL, 15 mL 02598R

S01EE05 TAFLUPROST Eye drops 15 micrograms per mL, single dose units 0.3 mL, 30 02748P

S01EE05 TAFLUPROST Eye drops 15 micrograms per mL, single dose units 0.3 mL, 30 02755B

S01ED02 BETAXOLOL Eye drops, solution, 5 mg (as hydrochloride) per mL, 5 mL 02825Q

S01EC54 BRINZOLAMIDE + TIMOLOL Eye drops 10 mg brinzolamide with timolol 5 mg (as maleate) per mL, 5 mL 03438Y

S01EA05 BRIMONIDINE Eye drops containing brimonidine tartrate 1.5 mg per mL, 5 mL 05298W

S01EA05 BRIMONIDINE Eye drops containing brimonidine tartrate 2 mg per mL, 5 mL 05534G

S01EA BRIMONIDINE + TIMOLOL Eye drops containing brimonidine tartrate 2 mg with timolol 5 mg (as maleate) per mL, 5 mL 05535H

S01EB01 PILOCARPINE Eye drops containing pilocarpine hydrochloride 10 mg per mL, 15 mL 05536J

S01EB01 PILOCARPINE Eye drops containing pilocarpine hydrochloride 20 mg per mL, 15 mL 05537K

S01EB01 PILOCARPINE Eye drops containing pilocarpine hydrochloride 40 mg per mL, 15 mL 05538L

S01EC04 BRINZOLAMIDE Eye drops 10 mg per mL, 5 mL 05540N

S01EC03 DORZOLAMIDE Eye drops 20 mg (as hydrochloride) per mL, 5 mL 05541P

S01EC DORZOLAMIDE + TIMOLOL Eye drops containing dorzolamide 20 mg (as hydrochloride) with timolol 5 mg (as maleate) per mL, 5 mL 05542Q

S01ED02 BETAXOLOL Eye drops, solution, 5 mg (as hydrochloride) per mL, 5 mL 05544T

S01ED01 TIMOLOL Eye drops 5 mg (as maleate) per mL, 5 mL 05548B

S01ED01 TIMOLOL Eye drops (gellan gum solution) 5 mg (as maleate) per mL, 2.5 mL 05550D

S01EE03 BIMATOPROST Eye drops 300 micrograms per mL, 3 mL 05551E

S01EE01 LATANOPROST Eye drops 50 micrograms per mL, 2.5 mL 05552F

S01EE LATANOPROST + TIMOLOL Eye drops 50 micrograms latanoprost with timolol 5 mg (as maleate) per mL, 2.5 mL 05553G

S01EE04 TRAVOPROST Eye drops 40 micrograms per mL, 2.5 mL 05554H

S01EE TRAVOPROST + TIMOLOL Eye drops 40 micrograms travoprost with timolol 5 mg (as maleate) per mL, 2.5 mL 05555J

S01EE BIMATOPROST + TIMOLOL Eye drops 300 micrograms bimatoprost with timolol 5 mg (as maleate) per mL, 3 mL 05558M

S01EC54 BRINZOLAMIDE + TIMOLOL Eye drops 10 mg brinzolamide with timolol 5 mg (as maleate) per mL, 5 mL 05562R

S01EA05 BRIMONIDINE Eye drops containing brimonidine tartrate 1.5 mg per mL, 5 mL 05563T

S01EA03 APRACLONIDINE Eye drops 5 mg (as hydrochloride) per mL, 10 mL 08083K

S01EE01 LATANOPROST Eye drops 50 micrograms per mL, 2.5 mL 08243W

S01EA05 BRIMONIDINE Eye drops containing brimonidine tartrate 2 mg per mL, 5 mL 08351M

S01EC04 BRINZOLAMIDE Eye drops 10 mg per mL, 5 mL 08483L

S01EC03 DORZOLAMIDE Eye drops 20 mg (as hydrochloride) per mL, 5 mL 08488R

S01EC DORZOLAMIDE + TIMOLOL Eye drops containing dorzolamide 20 mg (as hydrochloride) with timolol 5 mg (as maleate) per mL, 5 mL 08567X

S01EE04 TRAVOPROST Eye drops 40 micrograms per mL, 2.5 mL 08597L

S01EE03 BIMATOPROST Eye drops 300 micrograms per mL, 3 mL 08620Q

S01EA BRIMONIDINE + TIMOLOL Eye drops containing brimonidine tartrate 2 mg with timolol 5 mg (as maleate) per mL, 5 mL 08826M

S01EE LATANOPROST + TIMOLOL Eye drops 50 micrograms latanoprost with timolol 5 mg (as maleate) per mL, 2.5 mL 08895E

S01EE TRAVOPROST + TIMOLOL Eye drops 40 micrograms travoprost with timolol 5 mg (as maleate) per mL, 2.5 mL 09057Q

S01EE BIMATOPROST + TIMOLOL Eye drops 300 micrograms bimatoprost with timolol 5 mg (as maleate) per mL, 3 mL 09464D

S01EE03 BIMATOPROST Eye drops 300 micrograms per mL, single dose units 0.4 mL, 30 10046R

S01EE03 BIMATOPROST Eye drops 300 micrograms per mL, single dose units 0.4 mL, 30 10053D

S01EE BIMATOPROST + TIMOLOL Eye drops 300 micrograms bimatoprost with timolol 5 mg (as maleate) per mL, single dose units 0.4 mL, 30 10107Y

S01EE BIMATOPROST + TIMOLOL Eye drops 300 micrograms bimatoprost with timolol 5 mg (as maleate) per mL, single dose units 0.4 mL, 30 10108B

S01EC54 BRINZOLAMIDE + BRIMONIDINE Eye drops 10 mg brinzolamide with 2 mg brimonidine tartrate per mL, 5 mL 10536M

S01EC54 BRINZOLAMIDE + BRIMONIDINE Eye drops 10 mg brinzolamide with 2 mg brimonidine tartrate per mL, 5 mL 10547D

S01ED01 TIMOLOL Eye drops (gellan gum solution) 5 mg (as maleate) per mL, 2.5 mL (S19A) 14241M

S01ED01 TIMOLOL Eye drops (gellan gum solution) 5 mg (as maleate) per mL, 2.5 mL (S19A) 14301Q

S01EE03 BIMATOPROST Eye drops 300 micrograms per mL, 3 mL 14315K

S01EE TRAVOPROST + TIMOLOL Eye drops 40 micrograms travoprost with timolol 5 mg (as maleate) per mL, 2.5 mL 14316L

S01EE BIMATOPROST + TIMOLOL Eye drops 300 micrograms bimatoprost with timolol 5 mg (as maleate) per mL, 3 mL 14317M

S01EC04 BRINZOLAMIDE Eye drops 10 mg per mL, 5 mL 14321R

S01EE LATANOPROST + TIMOLOL Eye drops 50 micrograms latanoprost with timolol 5 mg (as maleate) per mL, 2.5 mL 14350G

S01EE BIMATOPROST + TIMOLOL Eye drops 300 micrograms bimatoprost with timolol 5 mg (as maleate) per mL, single dose units 0.4 mL, 30 14351H

S01EB01 PILOCARPINE Eye drops containing pilocarpine hydrochloride 10 mg per mL, 15 mL 14355M

S01EC DORZOLAMIDE + TIMOLOL Eye drops containing dorzolamide 20 mg (as hydrochloride) with timolol 5 mg (as maleate) per mL, 5 mL 14386E

S01EE03 BIMATOPROST Eye drops 300 micrograms per mL, single dose units 0.4 mL, 30 14422C

S01EC54 BRINZOLAMIDE + BRIMONIDINE Eye drops 10 mg brinzolamide with 2 mg brimonidine tartrate per mL, 5 mL 14423D

S01ED02 BETAXOLOL Eye drops, solution, 5 mg (as hydrochloride) per mL, 5 mL 14425F

S01EE01 LATANOPROST Eye drops 50 micrograms per mL, 2.5 mL 14453Q

S01EA BRIMONIDINE + TIMOLOL Eye drops containing brimonidine tartrate 2 mg with timolol 5 mg (as maleate) per mL, 5 mL 14491Q

S01EC54 BRINZOLAMIDE + TIMOLOL Eye drops 10 mg brinzolamide with timolol 5 mg (as maleate) per mL, 5 mL 14495X

S01EA05 BRIMONIDINE Eye drops containing brimonidine tartrate 1.5 mg per mL, 5 mL 14496Y

S01EA05 BRIMONIDINE Eye drops containing brimonidine tartrate 2 mg per mL, 5 mL 14497B

S01EB01 PILOCARPINE Eye drops containing pilocarpine hydrochloride 20 mg per mL, 15 mL 14523J

S01EC03 DORZOLAMIDE Eye drops 20 mg (as hydrochloride) per mL, 5 mL 14524K

S01EE04 TRAVOPROST Eye drops 40 micrograms per mL, 2.5 mL 14549R

S01EB01 PILOCARPINE Eye drops containing pilocarpine hydrochloride 40 mg per mL, 15 mL 14550T

S01ED01 TIMOLOL Eye drops (gellan gum solution) 5 mg (as maleate) per mL, 2.5 mL - (Timoptol-LA) (S19A) 14611B

S01ED01 TIMOLOL Eye drops (gellan gum solution) 5 mg (as maleate) per mL, 2.5 mL - (Timoptol-LA) (S19A) 14634F

S01ED01 TIMOLOL Eye drops 5 mg (as maleate) per mL, 5 mL (S19A) 14649B

S01ED01 TIMOLOL Eye drops 5 mg (as maleate) per mL, 5 mL (S19A) 14657K

Appendix 2: Table extracted from TGA Medicines Shortages Archive Result Summary for timolol-containing eye drops from January 2020 to August 2025.

| ARTG name | Active ingredients | Supply impact start date | Supply impact end date | Deletion from market |
| --- | --- | --- | --- | --- |
| TIMOPTOL-XE timolol 5mg/mL (as maleate) eye drops | timolol maleate | 20/04/2020 | 10/07/2020 |  |
| XALAMOL 50/5 latanoprost 50 microgram/mL + timolol (as maleate) 5 mg/mL eye drops bottle | timolol maleate~latanoprost | 2/06/2020 | 31/07/2020 |  |
| Latanoprost/Timolol Sandoz 50/5 latanoprost 50 microgram/mL timolol 5 mg/mL Eye Drops | latanoprost~timolol maleate | 1/07/2020 |  | 1/07/2020 |
| APO-DORZOLAMIDE/TIMOLOL 20/5 dorzolamide/timolol eye drops | Dorzolamide hydrochloride~Timolol maleate | 11/08/2020 |  | 11/08/2020 |
| COSDOR dorzolamide (as hydrochloride) 20 mg/mL and timolol (as maleate) 5 mg/mL eye drop bottle | timolol maleate~dorzolamide hydrochloride | 12/03/2021 | 18/06/2021 |  |
| AZARGA brinzolamide 1% and timolol 0.5% eye drops. | timolol maleate~brinzolamide | 22/11/2021 | 2/12/2021 |  |
| APO-Latanoprost/Timolol 0.05/5 Eye drops latanoprost 50 ug/mL and timolol (as maleate) 5 mg/mL bottle | timolol maleate~latanoprost | 21/01/2022 | 15/04/2022 |  |
| Timoptol 0.5% Eye Drops Bottle | timolol maleate | 11/04/2022 | 6/05/2022 |  |
| XALAMOL 50/5 latanoprost 50 microgram/mL + timolol (as maleate) 5 mg/mL eye drops bottle | timolol maleate~latanoprost | 29/04/2022 | 9/02/2023 |  |
| Timoptol 0.5% Eye Drops Bottle | timolol maleate | 28/06/2022 | 28/06/2022 |  |
| VIZO-PF DORZOLATIM dorzolamide (as hydrochloride) 20 mg/mL and timolol (as maleate) 5 mg/mL eye drop solution multi-dose bottle | timolol maleate~dorzolamide hydrochloride | 15/12/2022 | 29/06/2023 |  |
| APO-Latanoprost/Timolol 0.05/5 Eye drops latanoprost 50 ug/mL and timolol (as maleate) 5 mg/mL bottle | timolol maleate~latanoprost | 1/02/2023 | 1/02/2023 |  |
| COSOPT PRESERVATIVE-FREE dorzolamide (as hydrochloride) 20mg/mL and timolol (as maleate) 5mg/mL single dose eye drop ampoule | timolol maleate~dorzolamide hydrochloride | 7/03/2023 | 24/03/2023 |  |
| Timoptol 0.5% Eye Drops Bottle | timolol maleate | 10/03/2023 | 10/03/2023 |  |
| COSOPT PRESERVATIVE-FREE dorzolamide (as hydrochloride) 20mg/mL and timolol (as maleate) 5mg/mL single dose eye drop ampoule | timolol maleate~dorzolamide hydrochloride | 27/04/2023 | 27/04/2023 |  |
| COSOPT dorzolamide hydrochloride 20mg and timolol maleate 5mg per mL eye drop bottle | timolol maleate~dorzolamide hydrochloride | 27/04/2023 | 27/04/2023 |  |
| COSOPT PRESERVATIVE-FREE dorzolamide (as hydrochloride) 20mg/mL and timolol (as maleate) 5mg/mL single dose eye drop ampoule | timolol maleate~dorzolamide hydrochloride | 9/05/2023 | 9/05/2023 |  |
| COSDOR dorzolamide (as hydrochloride) 20 mg/mL and timolol (as maleate) 5 mg/mL eye drop bottle | timolol maleate~dorzolamide hydrochloride | 23/05/2023 | 17/08/2023 |  |
| COSOPT dorzolamide hydrochloride 20mg and timolol maleate 5mg per mL eye drop bottle | dorzolamide hydrochloride~timolol maleate | 22/06/2023 | 22/06/2023 |  |
| TIMOPTOL-XE timolol 5mg/mL (as maleate) eye drops | timolol maleate | 30/06/2023 | 30/06/2023 |  |
| Timoptol 0.5% Eye Drops Bottle | timolol maleate | 7/07/2023 | 7/07/2023 |  |
| TIMOPTOL-XE timolol 5mg/mL (as maleate) eye drops | timolol maleate | 22/08/2023 | 22/08/2023 |  |
| COSDOR dorzolamide (as hydrochloride) 20 mg/mL and timolol (as maleate) 5 mg/mL eye drop bottle | timolol maleate~dorzolamide hydrochloride | 2/11/2023 | 29/02/2024 |  |
| XALAMOL 50/5 latanoprost 50 microgram/mL + timolol (as maleate) 5 mg/mL eye drops bottle | timolol maleate~latanoprost | 14/11/2023 | 5/09/2024 |  |
| COSOPT PRESERVATIVE-FREE dorzolamide (as hydrochloride) 20mg/mL and timolol (as maleate) 5mg/mL single dose eye drop ampoule | dorzolamide hydrochloride~timolol maleate | 31/07/2024 |  | 31/07/2024 |
| COSOPT dorzolamide hydrochloride 20mg and timolol maleate 5mg per mL eye drop bottle | timolol maleate~dorzolamide hydrochloride | 5/08/2024 | 20/03/2025 |  |
| TIMOPTOL-XE timolol 5mg/mL (as maleate) eye drops | timolol maleate | 12/08/2024 | 11/06/2025 |  |
| Timoptol 0.5% Eye Drops Bottle | timolol maleate | 29/11/2024 | 27/05/2025 |  |
| APO-Latanoprost/Timolol 0.05/5 Eye drops latanoprost 50 ug/mL and timolol (as maleate) 5 mg/mL bottle | timolol maleate~latanoprost | 20/12/2024 | 4/04/2025 |  |
| VIZO-PF DORZOLATIM dorzolamide (as hydrochloride) 20 mg/mL and timolol (as maleate) 5 mg/mL eye drop solution multi-dose bottle | dorzolamide hydrochloride~timolol maleate | 30/04/2025 | 28/07/2025 |  |
